# An Immune Cell Signature Is Associated With Disease-Free Survival and Adjuvant Chemosensitivity of Patients With Resectable Gastric Cancer

**DOI:** 10.3389/fimmu.2020.621623

**Published:** 2021-02-04

**Authors:** Hongfei Yan, Yang Chen, Zichang Yang, Zhi Li, Xiaofang Che, Jiawen Xiao, Yunpeng Liu, Xiujuan Qu

**Affiliations:** ^1^ Department of Medical Oncology, The First Hospital of China Medical University, Shenyang, China; ^2^ Key Laboratory of Anticancer Drugs and Biotherapy of Liaoning Province, The First Hospital of China Medical University, Shenyang, China; ^3^ Liaoning Province Clinical Research Center for Cancer, The First Hospital of China Medical University, Shenyang, China; ^4^ Key Laboratory of Precision Diagnosis and Treatment of Gastrointestinal Tumors, Ministry of Education, The First Hospital of China Medical University, Shenyang, China; ^5^ Department of Respiratory and Infectious Disease of Geriatrics, The First Hospital of China Medical University, Shenyang, China; ^6^ Department of Medical Oncology, Shenyang Fifth People Hospital, Shenyang, China

**Keywords:** immunoscore, gastric cancer, adjuvant chemotherapy, DFS, XP

## Abstract

Increasing evidence has indicated that current tumor-node-metastasis (TNM) stage alone cannot predict prognosis and adjuvant chemotherapy benefits accurately for stages II and III gastric cancer (GC) patients after surgery. In order to improve the predictive ability of survival and adjuvant chemotherapy benefits of GC patients after surgery, this study aimed to establish an immune signature based on the composition of infiltrating immune cells. Twenty-eight types of immune cell fractions were evaluated based on the expression profiles of GC patients from the Gene Expression Omnibus (GEO) database using single-sample gene set enrichment analysis (ssGSEA). The immunoscore (IS) was constructed using a least absolute shrinkage and selection operator (LASSO) Cox regression model. Through the LASSO model, an IS classifier consisting of eight immune cells was constructed. Significant difference was found between high-IS and low-IS groups in the training cohort in disease-free survival (DFS, P < 0.0001) and overall survival (OS, P < 0.0001). Multivariate analysis showed that the IS classifier was an independent prognostic indicator. Moreover, a combination of IS and TNM stage exhibited better prognostic value than TNM stage alone. Further analysis demonstrated that low-IS patients who had more tumor-infiltrating lymphocytes had better response to adjuvant chemotherapy. More importantly, we found that patients with high-IS were more likely to benefit from a Xeloda plus cisplatin regimen after surgery. Finally, we established two nomograms to screen the stage II and III GC patients who benefitted from adjuvant chemotherapy after surgery. The combination of IS classifier and TNM stage could predict DFS and OS of GC patients. The IS model has been proven as a promising tool that can be used to identify the patients with stages II and III GC who may benefit from adjuvant chemotherapy.

## Introduction

Gastric cancer (GC) is the fifth most common malignant cancer and the third leading cause of cancer-related deaths around the world (1). Although treatments for GC have improved rapidly, for patients with resectable GC, as stage I, II, and partial III GC, surgical resection is the preferred treatment. As the rates of recurrence for GC patients following surgery range from 25% to 40% (2–5), adjuvant chemotherapy is important; however, much research has revealed variations in clinical outcomes in patients with similar treatments at the same TNM stage (5, 6), indicating the insufficient clinical information provided by TNM stage which is the most useful staging system for cancers in clinical practice (7–9). Although several studies have developed models to reinforce the prognostic ability of TNM stage (10, 11), most of which are established with the expression of proteins in cancer cells detected by immunohistochemistry based on the OS of patients (11, 12), ignoring the effects of tumor microenvironment (TME) and DFS.

The concept, immunoscore (IS), was proposed for use in analysis of colon cancer at the first time, consisting of two markers of cytotoxic and memory T cells (13). Consequently, studies have reported that the scores assessed by immune cell markers could predict recurrence, DFS and OS of patients with stage I-IV colon cancer (14, 15). Moreover, a recent investigation demonstrated the predictive value of IS in oxaliplatin-based adjuvant chemotherapy. The International Duration Evaluation of Adjuvant Therapy (IDEA) France cohort study showed that 3-year DFS rates of patients who received mFOLFOX6 for 6 months were not dramatically superior to those treated for 3 months (72% *vs*. 76%; HR, 1.44; 95% CI, 1.14–1.82) (16), however, a follow-up study on IS, consisting of CD3 and CD8, revealed that patients whose tumor had been infiltrated by more lymphocytes (IS-Intermediate+ High) benefited more from 6 months of oxaliplatin-based adjuvant chemotherapy compared with those treated with 3 months of adjuvant chemotherapy (HR, 0.528; 95% CI, 0.372-0.750; *P* = 0.0004), indicating that tumor-infiltrating immune cells (TIICs) have critical effects on DFS and adjuvant chemosensitivity in cancers (17).

Like colon cancer, adjuvant chemotherapy has been verified to improve DFS and OS compared with surgery alone in GC in many studies (5, 18–20). Results of CLASSIC trial showed that the 5-year DFS in adjuvant chemotherapy group was 68% *versus* 53% in the observation group though the median OS was only a moderate benefit of 9% (5), indicating that adjuvant chemotherapy could significantly improve DFS of GC patients; however, subgroup analysis of the Adjuvant Chemotherapy Trial of TS-1 Gastric Cancer (ACTS-GC) revealed that adjuvant chemotherapy did not improve OS in stage III patients compared with observation alone, but conversely increased adverse effects (21). Therefore, it is necessary to improve patient selection for adjuvant chemotherapy and outcome prediction for individual treatment. Recent studies have developed models with protein markers in GC cells detected by immunohistochemistry (10, 22). However, in these studies, tumor microenvironment (TME) was ignored, which plays a critical role during tumorigenesis, progression, and therapeutic efficacy (23–25).

In this study, we described the landscape of 28 immune cells in GC applying ssGSEA and established a novel immune cells-based model using LASSO Cox regression, IS, to predict DFS and OS in patients after surgery. Moreover, the model could select the patients who might benefit from adjuvant chemotherapy. To apply the IS classifier to clinical practice, we constructed two nomograms to screen benefit population for adjuvant chemotherapy with accurate clinicopathological risk factors.

## Materials and Methods

### Patients Selection and Data Collection

**Figure 1:**
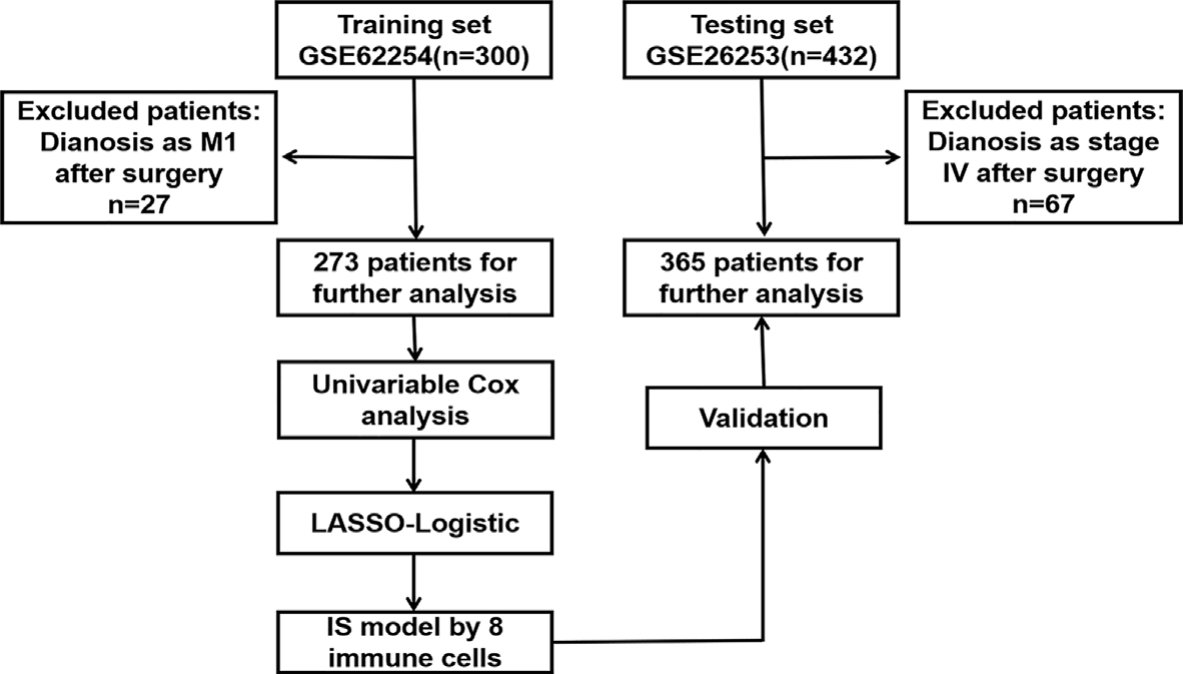
The flowchart through the identification procedure and analyses. *LASSO* least absolute shrinkage and selection operator.

Microarray dataset GSE62254, used as a training cohort for its complete clinicopathological and survival information, was downloaded from GEO database (https://www.ncbi.nlm.nih.gov/geo/). GSE26253 dataset, which included 432 GC patients with complete survival information, was used as a testing cohort. The inclusion and exclusion criteria were exhibited in **Figure 1**. The patients diagnosed as IV stage after surgery were filtered by the criteria since DFS was the main endpoint in this study.

### Estimation of Immune Cell Type Fractions

Gene expression profiles of GSE62254 and GSE26253 datasets downloaded from GEO database were analyzed by ssGSEA, which classifies gene sets with common physiological regulation, chromosomal localization and biological functions (26). Gene markers of 28 immune cells [activated CD4^+^ T cells (CD4^+^ Ta), activated CD8^+^ T cells (CD8^+^ Ta), activated dendritic cells (aDC), CD56^bright^ natural killer cells (CD56^+^ NK), CD56^dim^ natural killer cells (CD56^−^ NK), activated B cells (Ba), central memory CD4^+^ T cells (CD4^+^ Tcm), central memory CD8^+^ T cells (CD8^+^ Tcm), effector memory CD4^+^ T cells (CD4^+^ Tem), effector memory CD8^+^ T cells (CD8^+^ Tem), eosinophils, gamma delta T cells (γδT), immature B cells (Bi), immature dendritic cells (DCi), mast cells, myeloid-derived suppressor cells (MDSC), memory B cells (Bm), monocytes, natural killer cells (NK), natural killer T cells (NK T), neutrophils, plasmacytoid dendritic cells (pDC), macrophages, regulatory T cells (Tregs), follicular helper T cells (Tfh), type-1 T helper cells (Th1), type-17 T helper cells (Th17), and type-2 T helper cells (Th2)] were obtained from a previous study (27). On the basis of the expression of these markers, the infiltration levels of immune cell types were quantified by ssGSEA in the R package GSVA (28).

### The Construction of Immunoscore Using LASSO Algorithm

To select the most useful prognostic immune cells, the “glmnet” package in R was utilized to perform the COX regression analysis with LASSO algorithm (29). Eight immune cells were selected from candidate cells by LASSO algorithm, where the data were subsampled and the tuning parameters were determined according to the expected generalization error estimated from 10-fold cross-validation. The optimal cut-off values were evaluated based on the association between DFS and cell fraction in the training cohort using the “survminer” package, dividing the patients into low-IS and high-IS groups.

### Construction of Nomograms

Following the multivariate Cox regression analysis for the selection of independent prognostic factors, IS and other clinical pathological characteristics were used to generate the nomograms and calibration plots by “rms” package in R. In this model, each factor was assigned a weight score based on the results of the multivariate Cox regression analysis. Calibration was used to assess the performance of the nomograms. Receiver operating characteristic (ROC) analysis was also performed to estimate the accuracy of the nomograms for survival prediction using the “survival ROC” package of R. In addition, C-index was calculated with “survival” package.

### Statistical Analysis

Group comparisons were performed for continuous and categorical variables using one-way ANOVA and the χ^2^ test. Survival curves were constructed by the Kaplan–Meier method and compared by means of the log rank test. Hazard ratios for univariable analyses were calculated using a univariable Cox proportional hazards regression model. A multivariable Cox regression model with the enter method was used to determine independent prognostic factors. Correlations between the immunoscore and mRNA expression of genes were analyzed by means of Pearson’s correlation test. The sensitivity and specificity of the survival prediction based on the immunoscore were depicted by a time-dependent receiver operating characteristic (ROC) curve, with quantification of the area under the ROC curve using the “timeROC” package. All statistical tests were two-sided and P < 0.050 was considered statistically significant. Statistical analyses were conducted using R software and SPSS^®^ version 19.0 (IBM, Armonk, New York, USA).

## Results

### Patient Characteristics

As shown by the flowchart (**Figure 1**), 273 patients from the GSE62254 dataset and 365 patients from the GSE26253 dataset with DFS and OS information were included after applying the exclusion criteria. The patients’ clinicopathological characteristics are detailed in **Table 1**. Of the 273 patients in the training cohort, 186 (68.1%) were men and the median age was 64 (28–84) years. In addition, the median DFS was 35.7 months and OS was 53.3 months. Of the 365 patients in the testing cohort, 239 (65.5%) were men and the median age was 53 (23–74) years. The median DFS and OS were 60.4 and 69.6 months, respectively, in the testing cohort. **Figure S1** shows the distribution of the immune cells in patients in the training cohort (**Figure S1A**) and the relationship between 28 TIICs (**Figure S1B**).

**Table 1:** Characteristics of patients in the training (GSE62254) and testing (GSE26253) cohorts.

Characteristics	GSE62254		GSE26253	
	Number of patients	Percentage (%)	Number of patients	Percentage (%)
Number of patients	273		365	
Gender				
Male	186	68.1	239	65.5
Female	87	31.9	126	34.5
Age				
≤60	104	38.1	276	75.6
>60	169	61.9	89	24.4
Depth of invasion				
T2	176	64.5		
T3+T4	97	35.5		
Lymph node metastasis				
N0	37	13.6		
N1+N2+N3	236	86.4		
TNM stage				
I	32	11.7	68	18.6
II	163	59.7	167	45.8
III	78	28.6	130	35.6
Lauren classification				
Intestinal	142	52	122	33.4
Diffuse	115	42.1	232	63.6
Mixed	16	5.9	11	3
Status of microsatellite				
MSS	206	75.5		
MSI	67	24.5		
Adjuvant Chemotherapy				
No	131	48		
Yes	142	52		

MSS, microsatellite stability; MSI, microsatellite instability.

### Construction of IS Model

**Figure 2:**
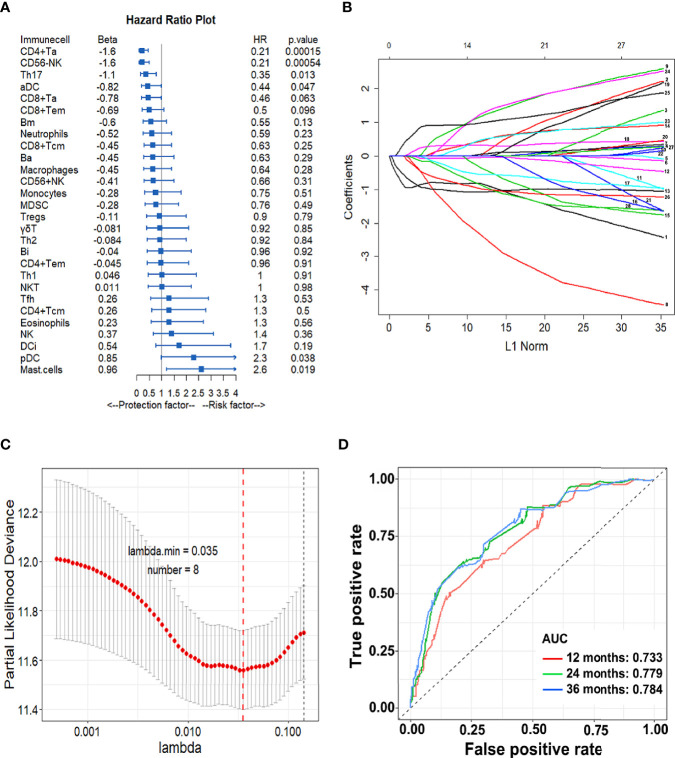
Construction of the IS model. **(A)** Forest plots of HRs for tumor infiltrating cells by univariate Cox analysis; **(B)** LASSO coefficient profiles of the 28 immune cell fractions; Immune cell types: 1.CD4^+^Ta; 2.CD8^+^Ta; 3.aDC; 4.CD56^+^ NK; 5.CD4^+^ Tcm; 6.CD8^+^ Tem; 7. CD4^+^ Tem; 8.CD8^+^Tcm; 9.NK; 10.NKT; 11.Th1; 12.Th17; 13.CD56^−^NK; 14.DCi; 15. Macrophages; 16.MDSC; 17.Neutrophils; 18.pDC; 19.Tregs; 20.Th2; 21.Ba; 22.Eosinophils; 23.γδT; 24.Bi; 25.Mast cells; 26.Bm; 27.Monocytes; 28.Tfh; **(C)** Ten-fold cross-validation for tuning parameter selection in the LASSO model. Error bars represent confidence intervals for partial likelihood deviance as *Λ* was changed. The dotted line indicates the optimal values; **(D)** The IS measured by time-dependent ROC curves in the training cohort. The area under the ROC curve is defined as AUC. *HR*, hazard ratio; *LASSO*, least absolute shrinkage and selection operator; *ROC*, receiver-operating characteristic.

Based on the relationship between 28 TIICs and DFS, we then constructed an immune-cell model. The forest plot in **Figure 2A** calculated by univariate Cox analysis shows the association between each immune cell subset and DFS. In general, CD4^+^ Ta (HR = 0.21, *P* < 0.001), aDC (HR = 0.44, *P *= 0.047), Th17 (HR = 0.35, *P* = 0.013), and CD56^-^ NK (HR = 0.21, *P* < 0.001) are protective factors for DFS. On the contrary, mast cell (HR = 2.6, *P* = 0.019) and pDC (HR = 2.3, *P* = 0.038) are risk factors. Then through LASSO Cox regression model eight immune cells were selected to build the IS and the formula was as follows: IS = 0.20810997 × DC + 0.79762920 × Mast cell - 0.8729771 × CD4^+^ Ta - 0.37309914 × CD8^+^ Tem - 0.04459008×Th17 - 0.88370283 × CD56^+^ NK - 0.12005145 × Ba-0.39656416×Bm (**Figures 2B, C**). We calculated an IS for each patient based on their personalized levels of the eight cells. The predictive accuracy of the model in the training cohort was assessed by time-dependent ROC analysis at 1, 2, and 3 years where AUC values were 0.733, 0.779, and 0.784, respectively (**Figure 2D**).

### IS and Prognosis

**Figure 3:**
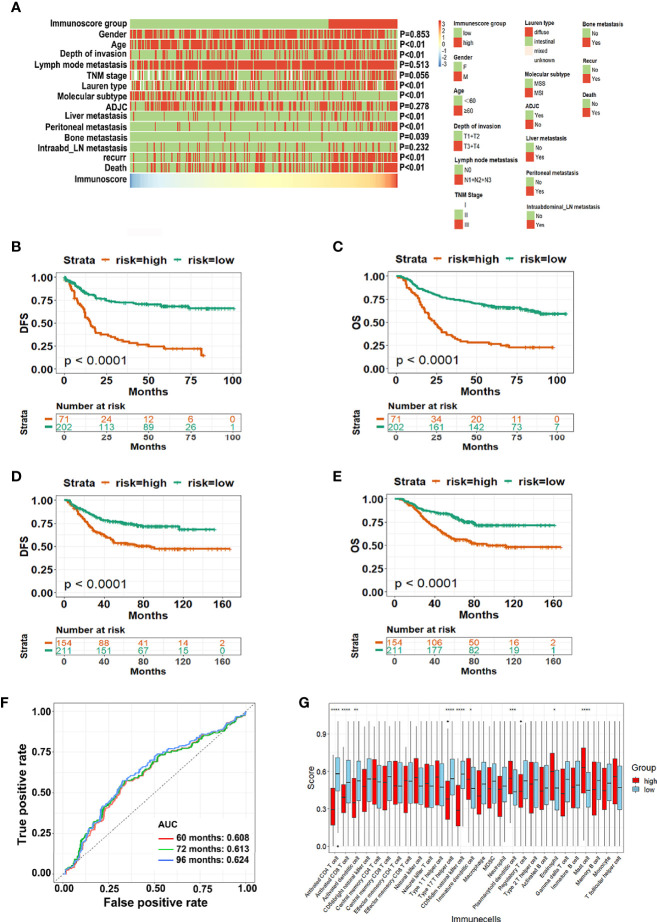
The predictive value of IS for GC patients with surgery. **(A)** Heatmaps summarizing the distribution of IS and clinical pathological characteristics in the training cohort; **(B, C)** Kaplan-Meier analysis for the DFS and OS of GC patients in the GSE62254 dataset; **(D, E)** Kaplan-Meier analysis for the DFS and OS of GC patients in the GSE26253 dataset; **(F)** The IS measured by time-dependent ROC curves in the testing cohort. The area under the ROC curve was defined as AUC; **(G)** Distribution of 28 immune cells transformed using ssGSEA in high-IS and low-IS groups. *ADJC*, adjuvant chemotherapy; *Intraabd_LN* intraabdominal lymph nodes; *DFS*, disease free survival; *OS*, overall survival; *GC*, gastric cancer; *ssGSEA* single-sample gene set enrichment analysis; *IS*, immunoscore; *ROC*, receiver-operating characteristic. *P < 0.05; **P < 0.01; ***P < 0.001; ****P < 0.0001.

Using the optimum cut-off value (IS = -0.65) obtained by the “survminer” package, patients in the training cohort were divided into high-IS and low-IS groups. **Figure 3A** shows the distribution of clinicopathological characteristics between high-IS and low-IS groups. The Kaplan-Meier curve in the training cohort revealed that patients in the low-IS group had longer DFS (*P* < 0.001) as well as OS (*P* < 0.001) compared with the high-IS group (**Figures 3B, C**). The 5-year DFS and OS for low-IS group were 28.2% and 60%, respectively and 14.1% and 22.5% respectively, for high-IS group. Similar results were observed in the testing cohort (*P* < 0.001 for both DFS and OS, **Figures 3D, E** and **Figure S2**). The ROC analysis of the testing cohort indicated that the model could predict the prognosis of GC patients accurately (**Figure 3F**). The multivariate Cox regression analysis showed that high-IS and TNM stage were independent prognostic indicators for both poor DFS and OS in either training cohort (**Table 2**, **Table S1**) or testing cohort (**Tables S2** and **S3**). Additionally, different distributions of immune cells between high-IS and low-IS groups are illustrated in **Figure 3G**. The infiltration of CD4^+^ Ta, CD8^+^ Ta, aDC, Th17, and CD56^-^ NK cells in the TME of low-IS patients was significantly greater than that in high-IS patients. These data demonstrated that the eight-immune cells model, IS, could precisely predict the DFS and OS of GC patients with surgery.

**Table 2:** Univariable and multivariable association of IS classifier, clinicopathological characteristics with DFS in the training cohort.

	Univariate analysis		Multivariate analysis	
	HR(95% CI)	P value	HR(95% CI)	P value
Gender	0.962(0.637–1.453)	0.855		
Age	1.005(0.987–1.023)	0.614		
T stage(T2+T3 vs. T1+T2)	2.602(1.772–3.822)	<0.01		
N stage(N1+N2+N3 vs. N0)	3.052(1.338–6.955)	0.008		
TNM stage(III vs. I+II)	3.744(2.386–5.875)	<0.01	2.279(1.278–4.055)	0.005
Lauren type	1.394(1.018–1.908)	0.038		
Status of microsatellite (MSI vs. MSS)	1.356(1.039–1.770)	0.025		
IS	1.794(1.492–2.158)	<0.01	1.581(1.279–1.954)	<0.01

MSS, microsatellite stability; MSI, microsatellite instability; HR, hazard ratio; IS, immunoscore.

We also assessed the relationships between IS, the status of relapse and survival, and the distribution of the eight cells in the training and testing cohorts. **Figure S3** shows that patients in the high-IS group had more recurrence and death events than among those in the low-IS group, which further verified the accuracy of the model.

### IS and TNM Stage

**Figure 4:**
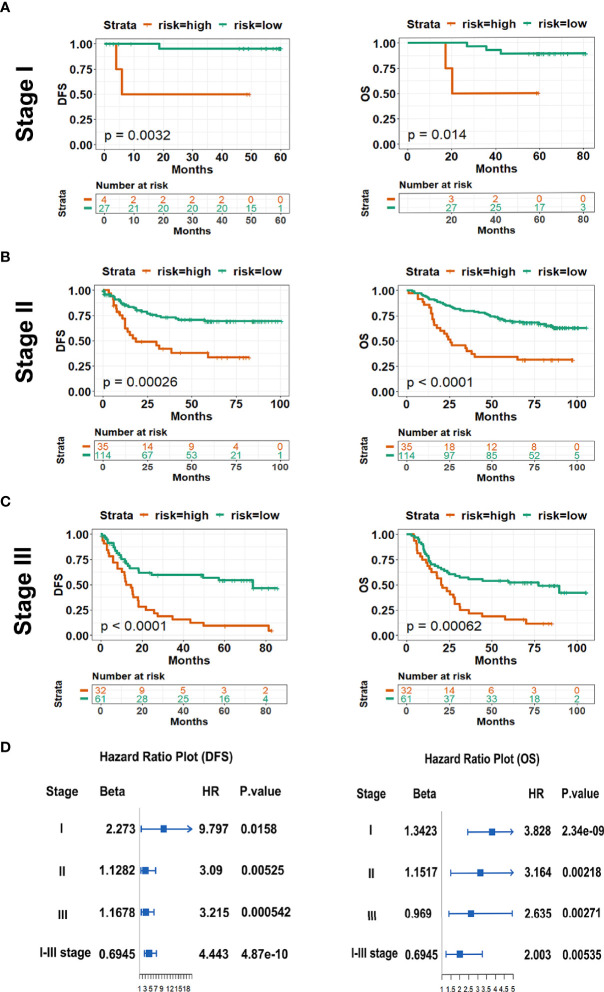
Kaplan-Meier survival analysis of DFS (left pane) and OS (right pane) according to IS in subgroups of patients with GC in the training cohort. **(A)** Stage I (*n* = 30); **(B)** Stage II (*n* = 97); **(C)** Stage III (*n* = 146); **(D)** The survival impact of IS in TNM stage subgroup. *HR*, hazard ratio; *IS*, immunoscore; *DFS*, disease free survival; *OS*, overall survival.

So far, the TNM stage has been regarded as a powerful indicator to predict the outcomes of patients with cancers, however, variable prognosis of patients was observed in clinical practice due to the heterogeneity. Here, to examine the value of the model, we respectively performed stratified analysis of the patients in stages I, II, and III in both the training and testing cohorts. Consistently, DFS and OS were all much longer in low-IS patients with stages I, II, and III GC than high-IS patients in either the training or testing cohort (**Figures 4A–C**, **Figures S4A–C**). Cox regression and forest plots were used to demonstrate that high-IS was a risk factor for both DFS and OS in stages I, II, and III (**Figure 4D**). Moreover, **Figure S5A** demonstrates that IS showed a better prognostic accuracy for DFS in GC patients with surgery than the TNM stage in the training cohort. Meanwhile, compared with the TNM stage alone, combining the data with IS showed a better prognostic value for DFS and OS in two cohorts (**Figure S5**). In conclusion, the prognostic value of IS is independent of TNM stage. For GC patients with surgery, the combination of IS and TNM stage for prediction of DFS and OS is superior to that of TNM alone.

### IS and the Subtype of GC

For GC patients, Lauren subtype and the status of microsatellite are the two recognized indicators to predict the outcomes and the efficacy of treatments. Therefore, we identified the value of IS based on Lauren classification and the status of microsatellite by using stratified analysis. **Figures S6A, B** show that low-IS patients had longer DFS (all *P* < 0.001 for intestinal and diffuse subtypes) and OS (*P* = 0.00046 for intestinal subtype, *P* < 0.001 for diffuse subtype, respectively) than high-IS patients. However, this phenomenon was not observed in mixed subtype patients (**Figure S6C**). Forest plots revealed that high-IS was the risk factor for DFS and OS simultaneously in both intestinal and diffuse subtypes (**Figure S6D**). Similarly, for patients with MSI, DFS and OS of high-IS patients were significantly shorter than those of low-IS patients (all *P* < 0.001, **Figure S7A**). Similar results were obtained in patients with microsatellite stability (MSS) on DFS (*P* < 0.001) and OS (*P* < 0.001) (**Figure S7B**). Meanwhile, high-IS was also a risk factor for DFS and OS regardless of the status of microsatellite stability (**Figure S7C**). Taken together, IS remains a statistically and clinically significant prognostic model when stratified by GC subtypes.

### IS and Adjuvant Chemotherapy

**Figure 5:**
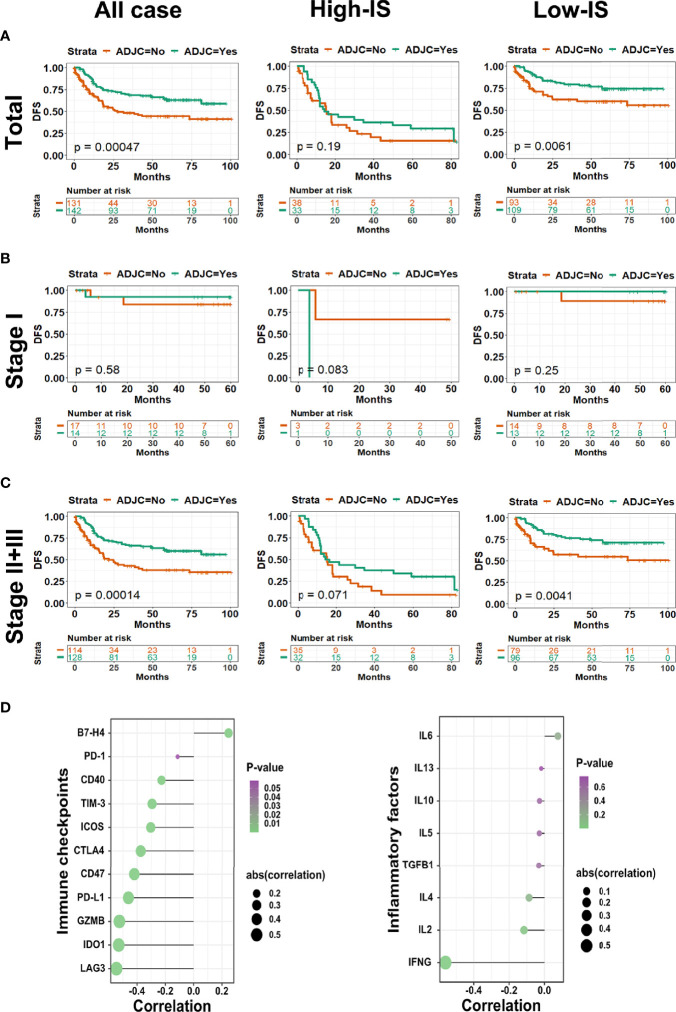
Comparing adjuvant chemotherapy benefit by DFS in total and TNM stage subgroups. Kaplan-Meier curves of **(A)** total cohort, **(B)** stage I and **(C)** stages II and III patients with GC stratified by the receipt of adjuvant chemotherapy; **(D)** Bubble diagrams describing the association between IS value and immune checkpoint regulators (left pane) and inflammatory cytokines (right pane). Bubble size represents the degree of correlation, bubble color denotes P-value. *DFS*, disease free survival; *GC*, gastric cancer.

For patients with surgery, especially stages II and III, adjuvant chemotherapy is indispensable. In the current study, the patients’ clinicopathological characteristics distributing in the group accepting adjuvant chemotherapy were similar to that in the group without chemotherapy after surgery (**Figure S8**). As shown in **Figure 5A**, adjuvant chemotherapy could improve DFS and OS simultaneously in the total cohort (P = 0.00047 for DFS). For patients in the low-IS group, adjuvant chemotherapy improved DFS (*P* = 0.0061), which was not observed in the high-IS group (*P* = 0.19; **Figure 5A**). The results from subset analysis in stage I showed no difference in DFS between patients receiving adjuvant chemotherapy and not receiving adjuvant chemotherapy (**Figure 5B**). However, the subset analysis in stages II and III patients demonstrated that adjuvant chemotherapy significantly improved DFS in the low-IS group (*P* = 0.0041), but no significant effect was found in the high-IS group (*P* = 0.071; **Figure 5C**). Stages II and III GC patients with low-IS would obtain longer DFS by receiving chemotherapy after surgery. To explain this phenomenon, we performed the relationship between IS value and the expression of immune checkpoint regulators and inflammatory mediators. Notably, IS value was shown to be negatively correlated with PD-L1 (*P* < 0.001, *r* = -0.19), CD40 (*P* < 0.001, *r* = -0.23), CD47 (*P *< 0.001, *r* = -0.42), CTLA4 (*P* < 0.001, *r* = -0.37), GZMB (*P* < 0.001, *r* = -0.52), Tim-3 (*P* < 0.001, *r* = -0.29), ICOS (*P* < 0.001, *r* = -0.30), IDO1 (*P* < 0.001, *r* = -0.53), LAG3 (*P* < 0.001, *r* = -0.55), and IFNG (*P* < 0.001, *r* = -0.56), whereas the interleukin family and TGF showed no statistical correlation (**Figure 5D**). Further, we compared the expression of these immune checkpoint regulators and inflammatory mediator between low-IS and high-IS groups. As **Figure S9** shown, the expression of PD-L1 (P<0.001), CD40 (P=0.001), CD47 (P<0.001), CTLA-4 (P<0.0001), GZMB (P<0.0001), Tim-3 (P<0.0001), ICOS (P<0.0001), IDO1 (P<0.0001), LAG3 (P<0.0001), and IFNG (P<0.0001) in low-IS group were all higher than that in high-IS group. In summary, these results indicated that IS could identify candidate stages II and III patients with surgery who would benefit from adjuvant chemotherapy.

**Figure 6:**
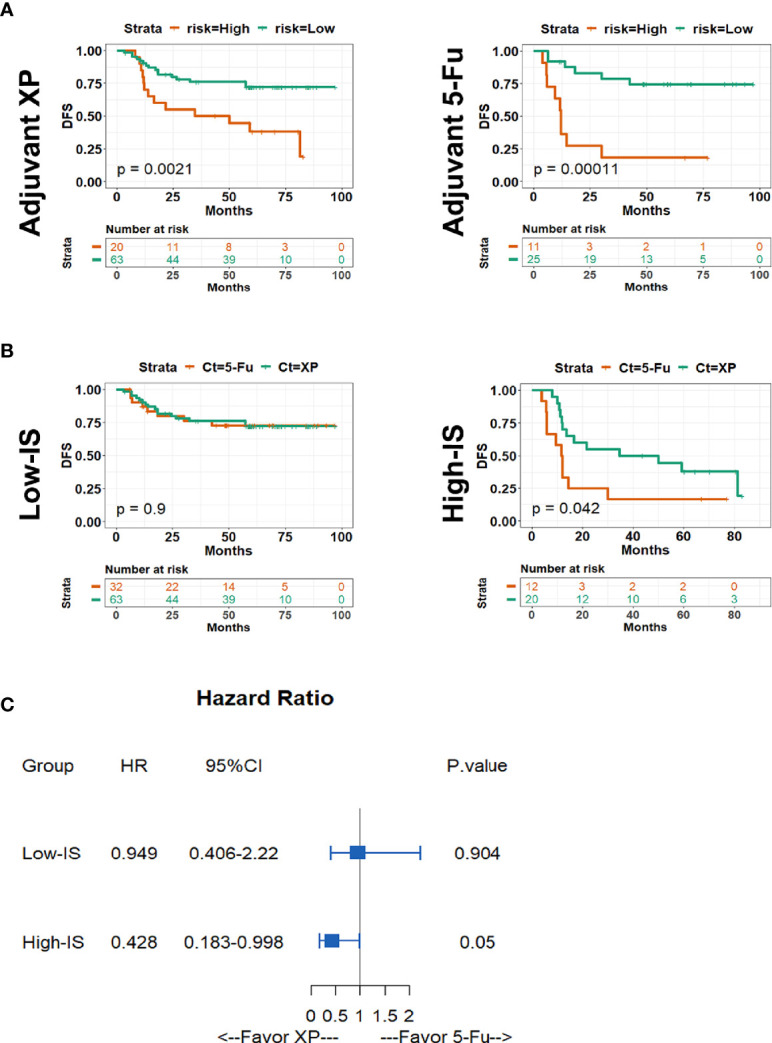
Kaplan-Meier survival analysis of DFS stratified by types of adjuvant chemotherapy among stages II and III patients. **(A)** Kaplan-Meier curves of patients in XP group (left panel) and 5-Fu group (right panel) stratified by IS; **(B)** Kaplan-Meier curves of patients in Low-IS group (left panel) and high-IS group (right panel) stratified by chemotherapy regimen; **(C)** Forest plot describing the benefit of chemotherapy regimen in different IS groups. *IS*, immunoscore; *XP*, Xeloda plus cisplatin; *5-Fu* 5-fluorouracil.

It is generally known that patients with stage II-III should receive adjuvant chemotherapy after surgery according to NCCN guidelines. Next, we investigated the effects of chemoregimen in the training dataset. Patients with stage II-III GC were divided into two groups, patients with Xeloda plus cisplatin (XP, 83 patients) and patients with chemoregimen based on fluorouracil (5-Fu, 44 patients), according to the adjuvant chemoregimen. As shown in **Figure 6A**, patients with low-IS had significantly longer DFS regardless of the adjuvant chemoregimen (*P* = 0.0021 and *P* = 0.00011 for the XP group and 5-Fu group, respectively). However, as patients were divided into low-IS and high-IS groups based on the IS classifier, we found that in high-IS group patients who received XP regimen after surgery had longer DFS compared with patients who received regimen 5-Fu (**Figure 6B**, *P* = 0.042), while in low-IS group patients there was no difference between two chemoregimen (**Figure 6B**, *P* = 0.9). Cox regression analysis notably demonstrated that patients with high-IS might benefit from XP rather than 5-Fu after surgery (**Figure 6C**).

**Figure 7:**
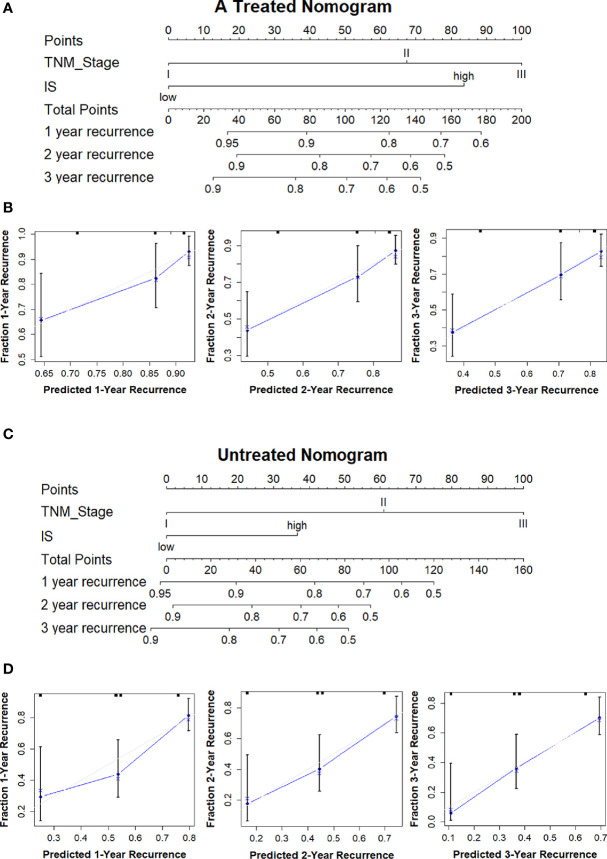
Treated and untreated nomograms to predict the probability of 1, 2, and 3-year recurrence with or without adjuvant chemotherapy in GC. **(A)** Treated nomogram predicting 1-, 2-, and 3-year DFS for GC patients after surgery with adjuvant chemotherapy; **(B)** Calibration curves to validate treated nomogram for 1-, 2-, and 3-year DFS; **(C)** Untreated nomogram predicting 1-, 2-, and 3-year DFS for GC patients after surgery without adjuvant chemotherapy; **(D)** Calibration curves to validate untreated nomogram for 1-, 2-, and 3-year DFS. *DFS*, disease free survival; *OS*, overall survival; *GC*, gastric cancer.

To provide a quantitative method in clinical practice to predict the probability of 1-, 2-, and 3-year DFS in patients with stages II and III GC after surgery, we established two nomograms integrating clinicopathological factors and IS on the basis of multivariate Cox regression analysis (**Figure 7**). The *c*-index values were 0.7 and 0.677 for the nomograms with adjuvant chemotherapy and without adjuvant chemotherapy after surgery respectively, indicating a satisfactory overlap with actual observations. The two nomograms based on IS could be used to predict the prognosis of patients with or without adjuvant chemotherapy in clinical practice.

## Discussion

In the present study, we analyzed the pattern of TIICs by using ssGSEA and constructed an eight-immune cells model through LASSO Cox regression based on DFS. In recent years, many immune score models have been generated to predict the prognosis and the therapeutic efficacy by immunohistochemical method (10, 22, 30); however, the results of immunohistochemistry would be limited by the small quantity of cell types and sample size. In our study, immune cells were assessed by high-throughput gene expression generated through ssGSEA which supplied an expanded view of immune contexts, allowing us to investigate more tumor subtypes with greater precision within a larger patient cohort. Moreover, other than previous studies which generated models mostly based on OS (11, 31, 32), our study mainly focused on the DFS of GC patients. Though OS is a recognized and widely used outcome measure for patients with tumors, its use as a marker of therapeutic benefit remains controversial (33, 34). Recurrence and metastasis are the two important factors shortening the OS of patients, indicating that it is essential to assess the relapse risk accurately to improve prognosis. In this study, our data demonstrated that patients in the low-IS group whose tumors are infiltrated more lymphocytes have longer DFS than those in the high-IS group and the same results were obtained in stages I, II, and III, respectively, by way of stratification analysis. Furthermore, ROC analysis showed that the predictive accuracy for DFS of this IS classifier was superior to TNM stage. In contrast with TNM staging system which is described based on anatomical characteristics, the IS model could provide the immunological information in the microenvironment of GC. According to our analysis, the combination of TNM stage and IS classifier had better predictive ability than TNM alone. In general, GC patients at the same TNM stage could be divided into different risk groups based on IS for receiving appropriate treatments to improve the outcome.

According to National Comprehensive Cancer Network (NCCN) guidelines and previous studies, adjuvant chemotherapy is regarded as a standard treatment for stage II and III patients (35). However, results of the ACTS-GC trial implied that adjuvant chemotherapy did not improve OS in stage III patients compared with observation alone, conversely it increased adverse effects (21). Therefore, many studies have been committed to identify low-risk patients with stage II and III GC who might not need adjuvant chemotherapy. In present study, the results demonstrated that DFS and OS of patients with low-IS were significantly longer than patients with high-IS, indicating that low-IS patients are more likely benefit from adjuvant chemotherapy. Previous research has found that TIICs are essential for chemotherapy response in various cancers (36), indicating that patients with more immune-cell infiltration are chemotherapy-sensitive (37). Consistently, our results stated that low-IS patients who are more likely to benefit from adjuvant chemotherapy had more activated CD4^+^ T cell, activated CD8^+^ T cell, aDC, Th17, and CD56^-^ NK cells infiltrating in their tumors. The underlying mechanisms might be that chemotherapy exerts an anti-tumor effect by triggering immunogenic cell death (ICD) beyond cytotoxicity *via* TIICs (37–39). The immune cells activated by chemotherapeutic agents secrete cytokines, such as interferon and interleukin, leading to the death of cancers. Coincidently, the results revealed that the expression of IFNG in the low-IS group was dramatically higher, indicating that IFNG might participate in the process of chemotherapy sensitization in low-IS GC patients whose tumors have more TIICs. Further studies were needed to investigate the underlying mechanisms of action between IS and chemosensitivity in GC.

Additionally, to further explore why there was no difference between patients receiving adjuvant chemotherapy or not in high-IS group, we analyzed subsets according to chemotherapy regimens. Interestingly, the results demonstrated that patients with high-IS are more likely to benefit from the XP regimen after surgery. Recent decade, studies have verified the advantage of postoperative chemotherapy in GC compared with surgery alone (4, 40). ACTS-GC and CLASSIC, two randomized phase III trials, showed that postoperative chemotherapy with S-1 or capecitabine plus oxaliplatin could decrease the risk of recurrence (5, 21). Similarly, the Intergroup 0116 (INT-0116) trial (4) and the Adjuvant Chemoradiation Therapy in Stomach Cancer (ARTIST) trial (2) demonstrated the efficacy of 5-Fu plus leucovorin (LV) and XP regimens in patients after surgery, however, little evidence has been supplied to compare these regimens to screen the patients who can benefit from each regimen. Similar to colon cancer that the MSI status is a biomarker to predict the lack of efficacy of adjuvant 5-Fu chemotherapy and recommend an adjuvant chemotherapy combining fluoropyrimidine and oxaliplatin for stage III patients (41), our IS classifier offers a method with which to select patients who benefit from XP and 5-Fu regimens respectively. Generally speaking, in our opinion patients with low-IS are always sensitive to adjuvant chemotherapy no matter the regimens after surgery. For patients with high-IS, we suggested XP regimen as their adjuvant chemotherapy after surgery.

In recent years, immune checkpoint inhibitors (ICIs) have opened a new era of immunotherapy in vary cancers, represented by anti-CTLA-4 and anti-PD (42–44). The concept of “immune checkpoints” was regarded as the important immune switch regulating the “activity” and “inhibitory” state of immune cells (45, 46). Increasing evidence has demonstrated that the expression of immune checkpoints genes could be used as predictive biomarkers for therapeutic response of ICIs (47, 48). In this study, we found a significantly negative correlation between IS value and several important immune checkpoint biomarkers. PD-L1 has been regarded as the most useful biomarker to predict the immunotherapeutic efficacy in clinical practice. Several studies have reported that PD-L1 expression could be induced by IFNγ as an exogenous regulation which is consistent with our result that low-IS patients with high expression of PD-L1 secreted more IFNγ (49). Therefore, we speculate that patients with low-IS might also benefit from immunotherapy, which warrants further investigation.

Our study has some limitations. Firstly, it was a retrospective study based on publicly available datasets, the potential bias inferring to unbalanced clinicopathological characteristics cannot be ignored. Secondly, due to the incompleteness of the information we obtained, it is possible that patients with immune system disorders or acute infection were included in our study, which ideally should have been excluded. Thus, further prospective studies are needed to validate our findings.

## Conclusions

In summary, the IS classifier is a novel prognostic tool based on the presence of eight immune cells that could significantly improve the prediction of recurrence and survival in GC patients with surgery. Moreover, the IS classifier is a useful model to identify patients who would be more likely to benefit from adjuvant chemotherapy. Stages II and III GC patients with low-IS could benefit from either 5-Fu or XP regimens as adjuvant chemotherapy, however, patients with high-IS are more sensitive to XP regimen. In conclusion, the IS might help make decisions that improve individual treatment regimes in clinical practice.

## Data Availability Statement

The datasets presented in this study can be found in online repositories. The names of the repository/repositories and accession number(s) can be found below: https://www.ncbi.nlm.nih.gov/geo/, 62254 https://www.ncbi.nlm.nih.gov/geo/, 26253.

## Author Contributions 

HY conceived and designed the study. HY, YC and ZY collected and analyzed the data. ZL and JX reviewed the methods. HY wrote the manuscript. XC, YL, and XQ reviewed the manuscript. All authors contributed to the article and approved the submitted version.

## Funding

This work was supported by the National Key Research and Development Program of China (2017YFC1308900), Technological Special Project of Liaoning Province of China (2019020176-JH1/103), Science and Technology Plan Project of Liaoning Province (NO.2013225585), The Key Research and Development Program of Liaoning Province (2018225060), The General Projects of Liaoning Province Colleges and Universities (LFWK201706), Science and Technology Plan Project of Shenyang city (19-112-4-099).

## Conflict of Interest

The authors declare that the research was conducted in the absence of any commercial or financial relationships that could be construed as a potential conflict of interest.

## Supplementary Material

Supplementary Figure 1 The distribution of tumor-infiltrating cells in GC patients in the training cohort. **(A)** The tumor-infiltrating cell composition profile for each GC sample. **(B)** The correlation between different infiltrating immune cells. *GC*, gastric cancer.

Supplementary Figure 2 Heatmap summarizing the correlation between IS and clinicalpathological characteristics in the testing cohort.

Supplementary Figure 3 IS analysis of GC patients. **(A)** IS distribution, status of recurrence and survival, distribution of eight immune cells of 273 training cohort patients. **(B)** IS distribution, status of recurrence and survival, distribution of eight immune cells of 365 testing cohort patients. *DFS*, disease free survival; *OS*, overall survival; *GC*, gastric cancer.

Supplementary Figure 4 Kaplan-Meier survival analysis of DFS (left pane) and OS (right pane) according to IS in TNM subgroups of patients with GC in the testing cohort. **(A)** Stage I (*n* = 68); **(B)** Stage II (*n* = 167); **(C)** Stage III (*n* = 130). *DFS*, disease free survival; *OS*, overall survival; *GC*, gastric cancer.

Supplementary Figure 5 ROC curves compare the prognostic accuracy of the IS with TNM in the training and testing cohorts. **(A)** Comparison of the accuracy of DFS by IS and TNM in the training and testing cohorts. **(B)** Comparison of the accuracy of OS by IS and TNM in the training and testing cohorts. *ROC*, receiver operator characteristic; *AUC*, area under curve.

Supplementary Figure 6 Kaplan-Meier survival analysis of DFS (left pane) and OS (right pane) according to IS in Lauren subtype of the training cohort. **(A)** Intestinal subtype (*n* = 142); **(B)** Diffuse subtype (*n* = 115); **(C)** Mixed subtype (*n *= 16); **(D)** The survival impact of IS in Lauren subtype. *HR*, hazard ratio; *DFS*, disease free survival; *OS*, overall survival.

Supplementary Figure 7 Kaplan-Meier survival analysis of DFS (left pane) and OS (right pane) according to IS in MSS/MSI subtypes of the training cohort. **(A)** MSI subtype (*n* = 67); **(B)** MSS subtype (*n* = 206); **(C)** The survival impact of IS in MSI/MSS subtypes. *HR*, hazard ratio; *MSS*, microsatellite stability; *MSI*, microsatellite instability; *DFS*, disease free survival; *OS* overall survival.

Supplementary Figure 8 Effects of adjuvant chemotherapy on DFS in different subgroups.

Supplementary Figure 9 PD-L1, CD40, CD47, CTLA4, GZMB, Tim-3, ICOS, IDO1, LAG3, and IFNG mRNA expression between the low- and high-IS groups. * *P* < 0.05
