# Supplementary material for: An Immune Cell Signature Is Associated With Disease-Free Survival and Adjuvant Chemosensitivity of Patients With Resectable Gastric Cancer
Source: Front Immunol. 2021 Feb 4;11:621623. doi: 10.3389/fimmu.2020.621623 (PMC7890018; doi:10.3389/fimmu.2020.621623)
Supplement: Supplementary Figure 1 — The distribution of tumor-infiltrating cells in GC patients in the training cohort. (A) The tumor-infiltrating cell composition profile for each GC sample. (B) The correlation between different infiltrating immune cells. GC, gastric cancer. [file DataSheet_1.pdf]

Figure S1

a.

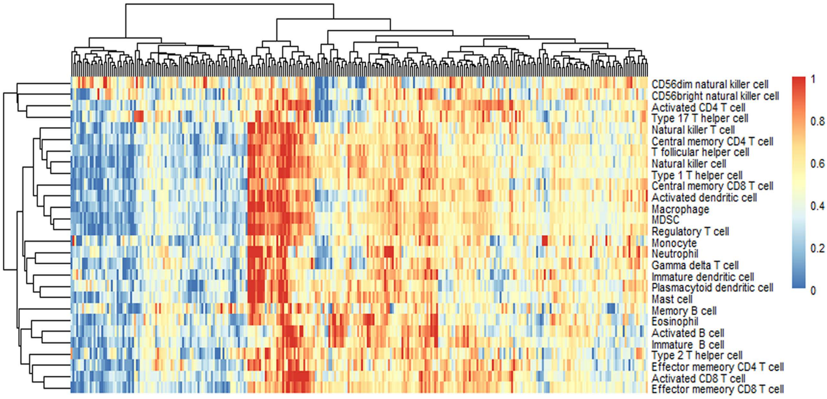

CD56dim natural killer cell
CD56bright natural killer cell
Activated CD4 T cell
Type 17 T helper cell
Natural killer T cell
Central memory CD4 T cell
T follicular helper cell
Natural killer cell
Type 1 T helper cell
Central memory CD8 T cell
Activated dendritic cell
Macrophage
MDSC
Regulatory T cell
Monocyte
Neutrophil
Gamma delta T cell
Immature dendritic cell
Plasmacytoid dendritic cell
Mast cell
Memory B cell
Eosinophil
Activated B cell
Immature B cell
Type 2 T helper cell
Effector memory CD4 T cell
Activated CD8 T cell
Effector memory CD8 T cell
1
0.8
0.6
0.4
0.2
0

b.

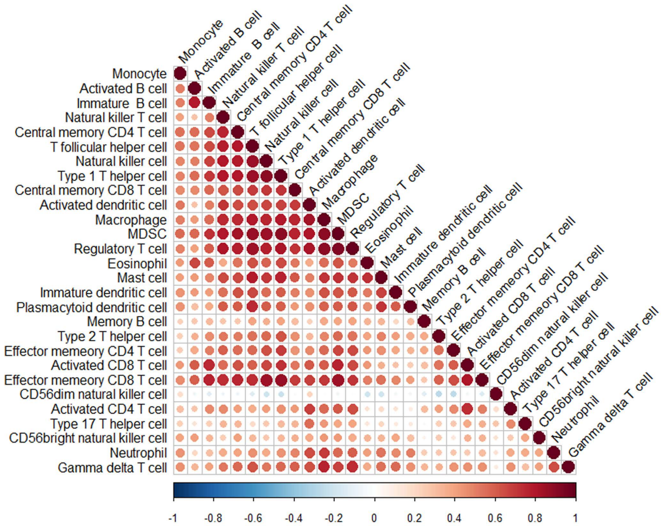

Monocyte
Activated B cell
Immature B cell
Natural killer T cell
Central memory CD4 T cell
T follicular helper cell
Natural killer cell
Type 1 T helper cell
Central memory CD8 T cell
Activated dendritic cell
Macrophage
MDSC
Regulatory T cell
Eosinophil
Mast cell
Immature dendritic cell
Plasmacytoid dendritic cell
Memory B cell
Type 2 T helper cell
Effector memeory CD4 T cell
Activated CD8 T cell
Effector memeory CD8 T cell
CD56dim natural killer cell
Activated CD4 T cell
Type 17 T helper cell
CD56bright natural killer cell
Neutrophil
Gamma delta T cell
-1
-0.8
-0.6
-0.4
-0.2
0
0.2
0.4
0.6
0.8
1

Figure S2

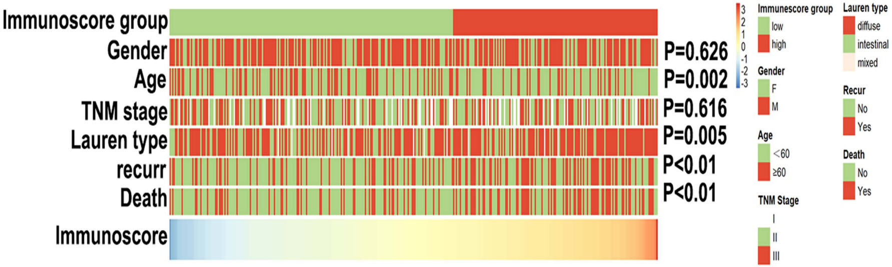
Immunoscore group
Gender
Age
TNM stage
Lauren type
recurr
Death
Immunoscore
P=0.626
P=0.002
P=0.616
P=0.005
P<0.01
P<0.01
3
2
1
0
-1
-2
-3
Immunescore group
low
high
Gender
F
M
Age
<60
≥60
TNM Stage
I
II
III
Lauren type
diffuse
intestinal
mixed
Recur
No
Yes
Death
No
Yes

Figure S3

a.

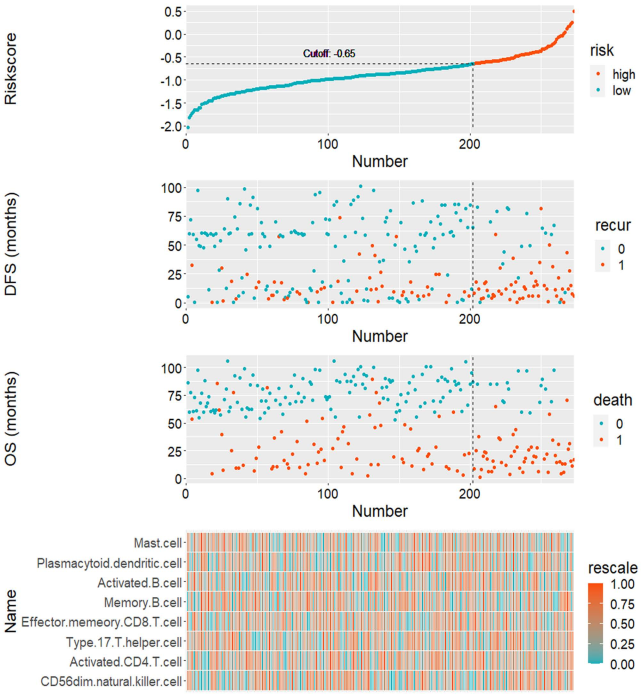
Riskscore
0.5
0.0
-0.5
-1.0
-1.5
-2.0
Cutoff: -0.65
0
100
200
Number
risk
high
low
DFS (months)
100
75
50
25
0
recur
0
1
OS (months)
death
Name
Mast.cell
Plasmacytoid.dendritic.cell
Activated.B.cell
Memory.B.cell
Effector.memeory.CD8.T.cell
Type.17.T.helper.cell
Activated.CD4.T.cell
CD56dim.natural.killer.cell
rescale
1.00
0.75
0.50
0.25
0.00

b.

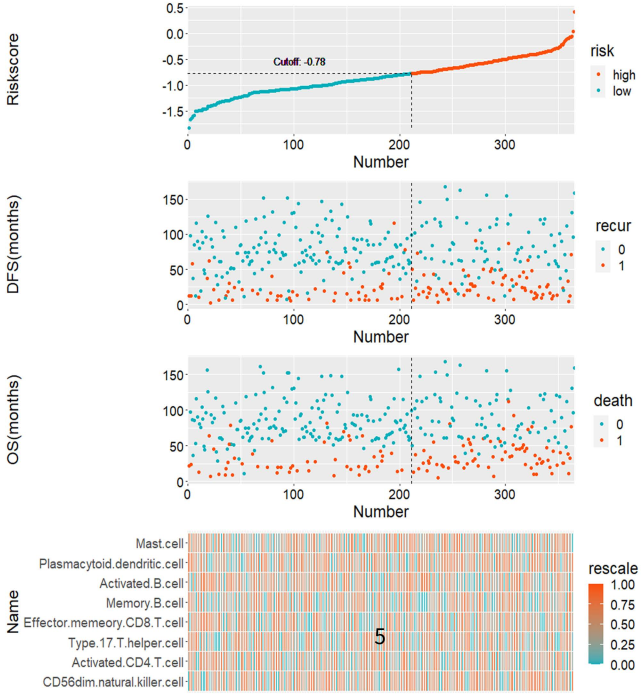
Riskscore
Cutoff: -0.78
0
100
200
300
Number
risk
high
low
DFS(months)
150
100
50
0
recur
0
1
OS(months)
death
Name
Mast.cell
Plasmacytoid.dendritic.cell
Activated.B.cell
Memory.B.cell
Effector.memeory.CD8.T.cell
Type.17.T.helper.cell
Activated.CD4.T.cell
CD56dim.natural.killer.cell
rescale
1.00
0.75
0.50
0.25
0.00

Figure S4

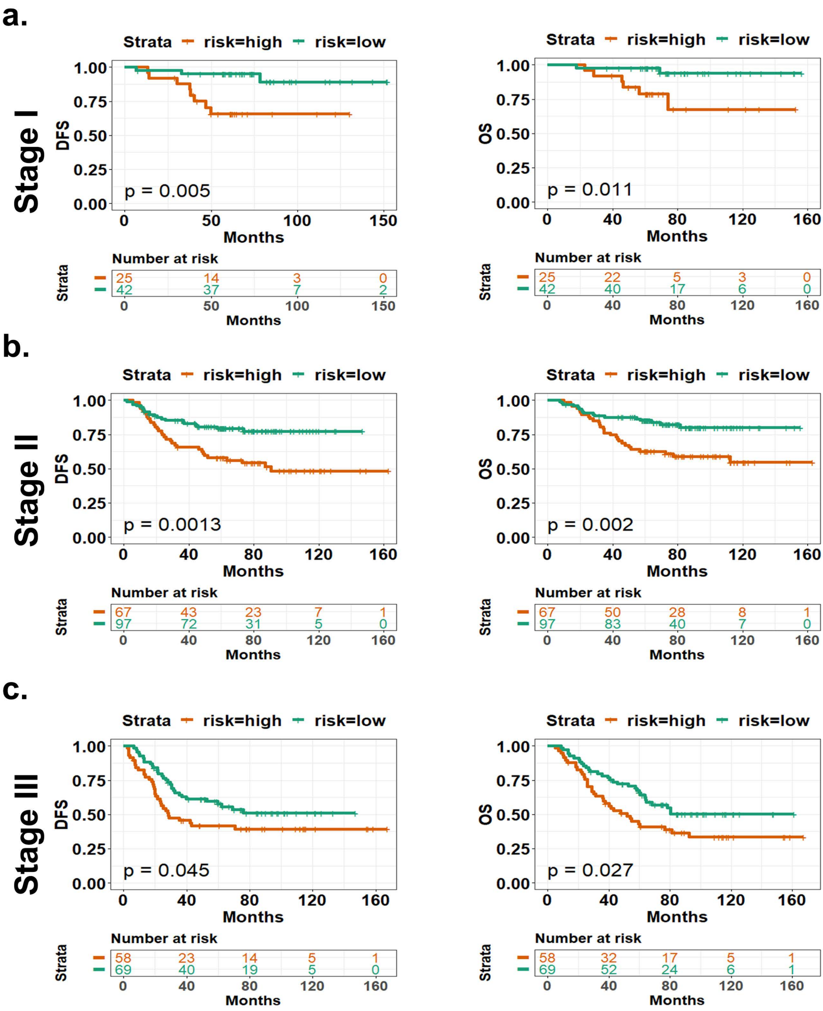

a.
Stage I
Strata risk=high risk=low
DFS
p = 0.005
OS
p = 0.011
Months
Number at risk
Strata
25 14 3 0
42 37 7 2
25 22 5 3 0
42 40 17 6 0
b.
Stage II
Strata risk=high risk=low
DFS
p = 0.0013
OS
p = 0.002
Months
Number at risk
Strata
67 43 23 7 1
97 72 31 5 0
67 50 28 8 1
97 83 40 7 0
c.
Stage III
Strata risk=high risk=low
DFS
p = 0.045
OS
p = 0.027
Months
Number at risk
Strata
58 23 14 5 1
69 40 19 5 0
58 32 17 5 1
69 52 24 6 1

Figure S5

a.

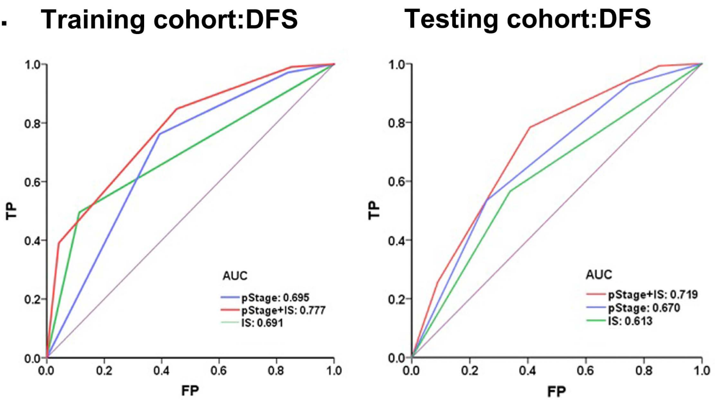
Training cohort:DFS
Testing cohort:DFS
TP
FP
1.0
0.8
0.6
0.4
0.2
0.0
AUC
pStage: 0.695
pStage+IS: 0.777
IS: 0.691
pStage+IS: 0.719
pStage: 0.670
IS: 0.613

b.

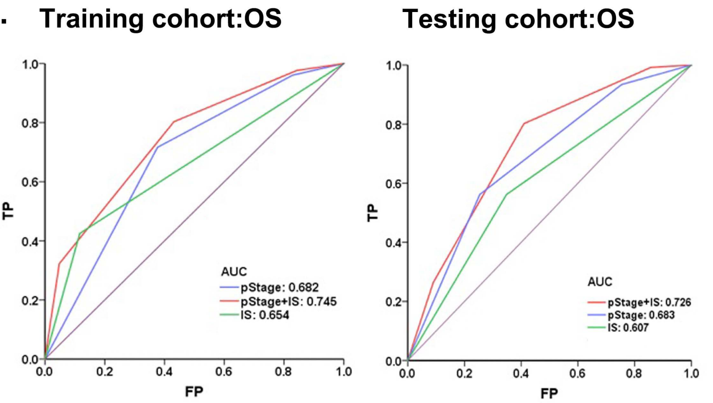
Training cohort:OS
Testing cohort:OS
TP
FP
1.0
0.8
0.6
0.4
0.2
0.0
AUC
pStage: 0.682
pStage+IS: 0.745
IS: 0.654
pStage+IS: 0.726
pStage: 0.683
IS: 0.607

Figure S6

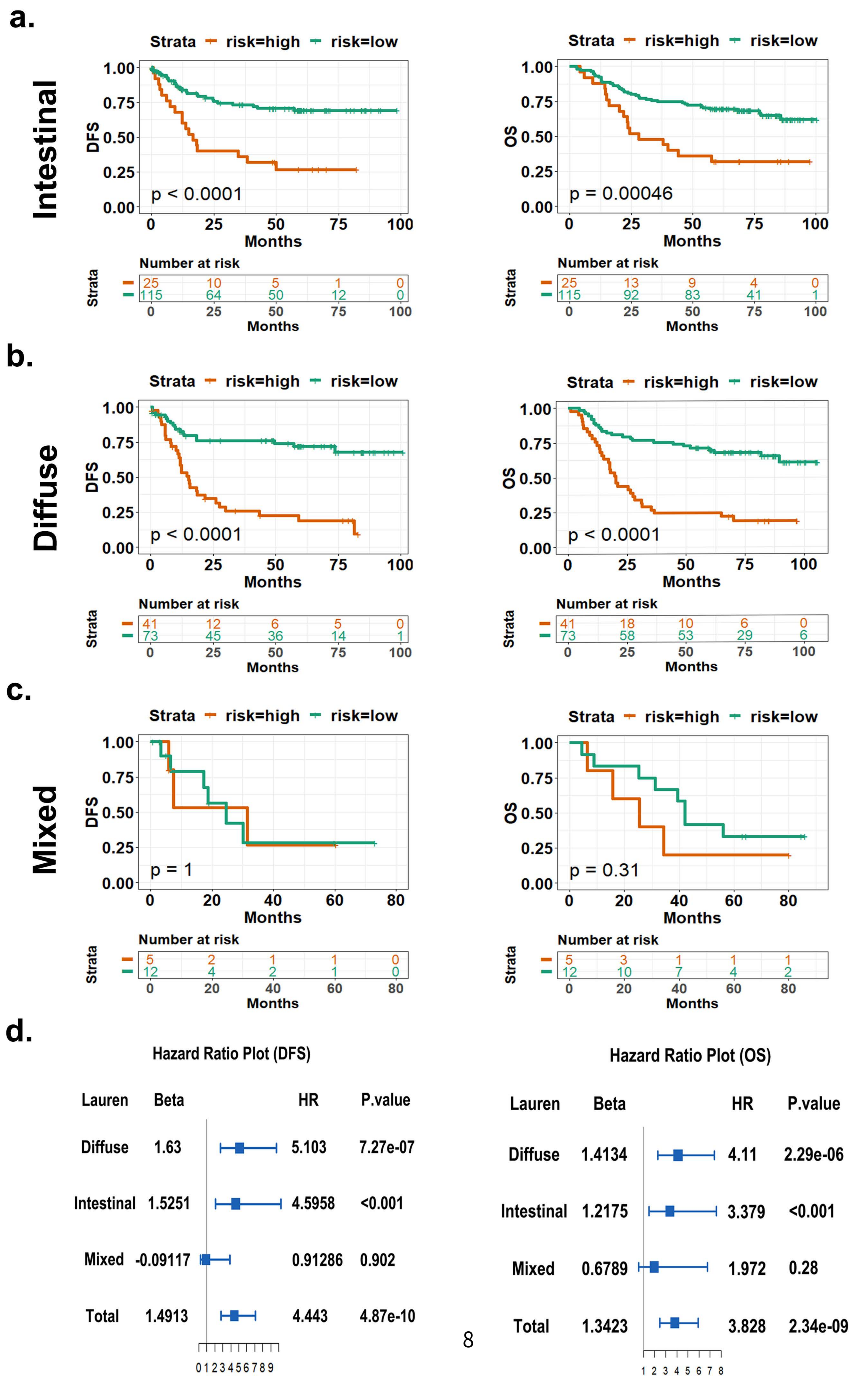
a.
b.
c.
d.
Intestinal
Diffuse
Mixed
Strata risk=high risk=low
DFS
OS
Months
Number at risk
p < 0.0001
p = 0.00046
p < 0.0001
p < 0.0001
p = 1
p = 0.31
Hazard Ratio Plot (DFS)
Lauren Beta HR P.value
Diffuse 1.63 5.103 7.27e-07
Intestinal 1.5251 4.5958 <0.001
Mixed -0.09117 0.91286 0.902
Total 1.4913 4.443 4.87e-10
Hazard Ratio Plot (OS)
Lauren Beta HR P.value
Diffuse 1.4134 4.11 2.29e-06
Intestinal 1.2175 3.379 <0.001
Mixed 0.6789 1.972 0.28
Total 1.3423 3.828 2.34e-09

Figure S7

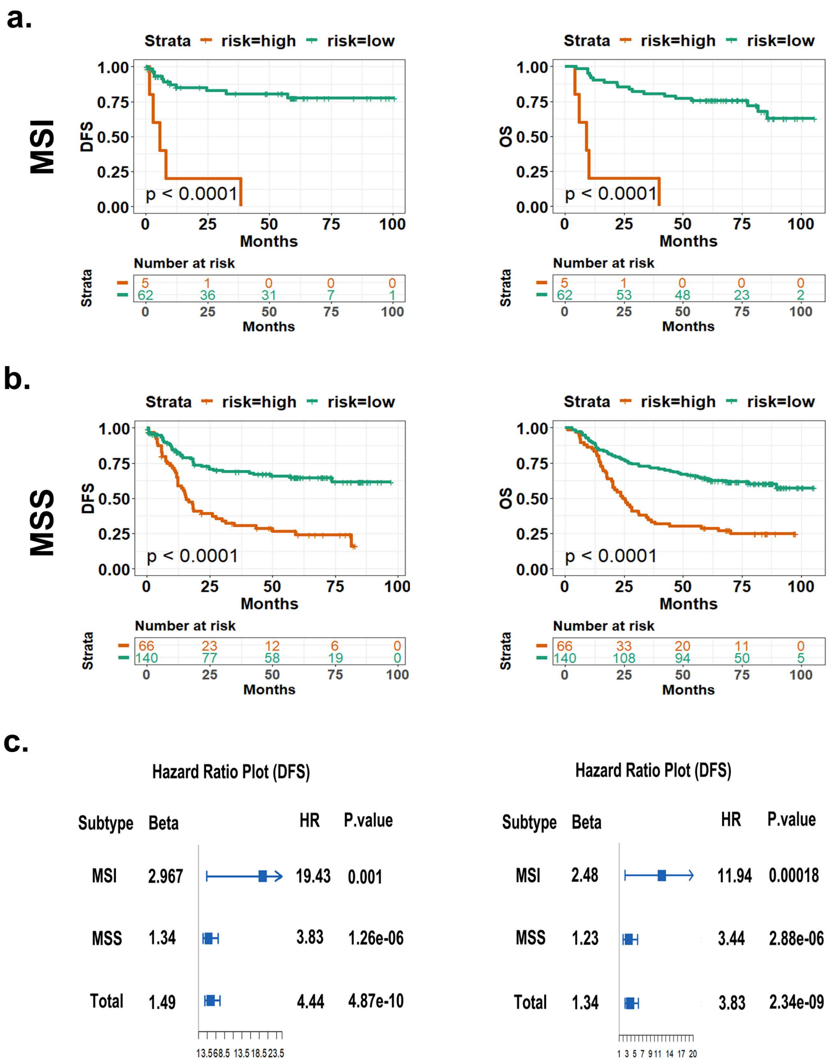

a.
MSI
Strata risk=high risk=low
DFS
OS
p < 0.0001
Months
Number at risk
Strata
5 1 0 0 0
62 36 31 7 1
5 1 0 0 0
62 53 48 23 2
b.
MSS
66 23 12 6 0
140 77 58 19 0
66 33 20 11 0
140 108 94 50 5
c.
Hazard Ratio Plot (DFS)
Subtype Beta HR P.value
MSI 2.967 19.43 0.001
MSS 1.34 3.83 1.26e-06
Total 1.49 4.44 4.87e-10
Hazard Ratio Plot (DFS)
Subtype Beta HR P.value
MSI 2.48 11.94 0.00018
MSS 1.23 3.44 2.88e-06
Total 1.34 3.83 2.34e-09

Figure S8

Hazard Ratio Plot (DFS)

Subgroup	Total	Chemo	No chemo	P-value	HR(95%CI)	P-value
IS	273	142	131	0.278		
Low	202	109	93		0.476(0.277-0.819)	0.007
High	71	33	38		0.692(0.398-1.203)	0.192
Stage I	30	14	16	0.351	0.470(0.043-5.193)	0.538
Low-IS	26	13	13		0.009(0-170072.102)	0.584
High-IS	4	1	3		434.450(0-8953863135600.588)	0.616
Stage II+III	243	128	115	0.389	0.485(0.328-0.717)	<0.001
Low-IS	176	96	80		0.469(0.271-0.810)	0.007
High-IS	67	32	35		0.598(0.339-1.054)	0.075
Depth of invasion	273	142	131	0.382		
T1+T2	176	95	81		0.587(0.335-1.030)	0.063
T3+T4	97	47	50		0.466(0.273-0.796)	0.005
Lymph metastasis	273	142	131	0.063		
N0	37	14	23		0.273(0.032-2.338)	0.236
N1+N2+N3	236	128	108		0.460(0.309-0.685)	<0.001

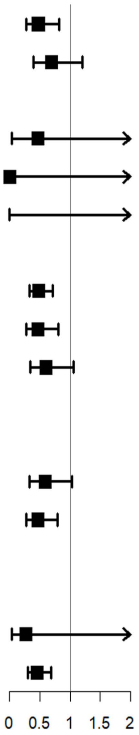

Figure S9

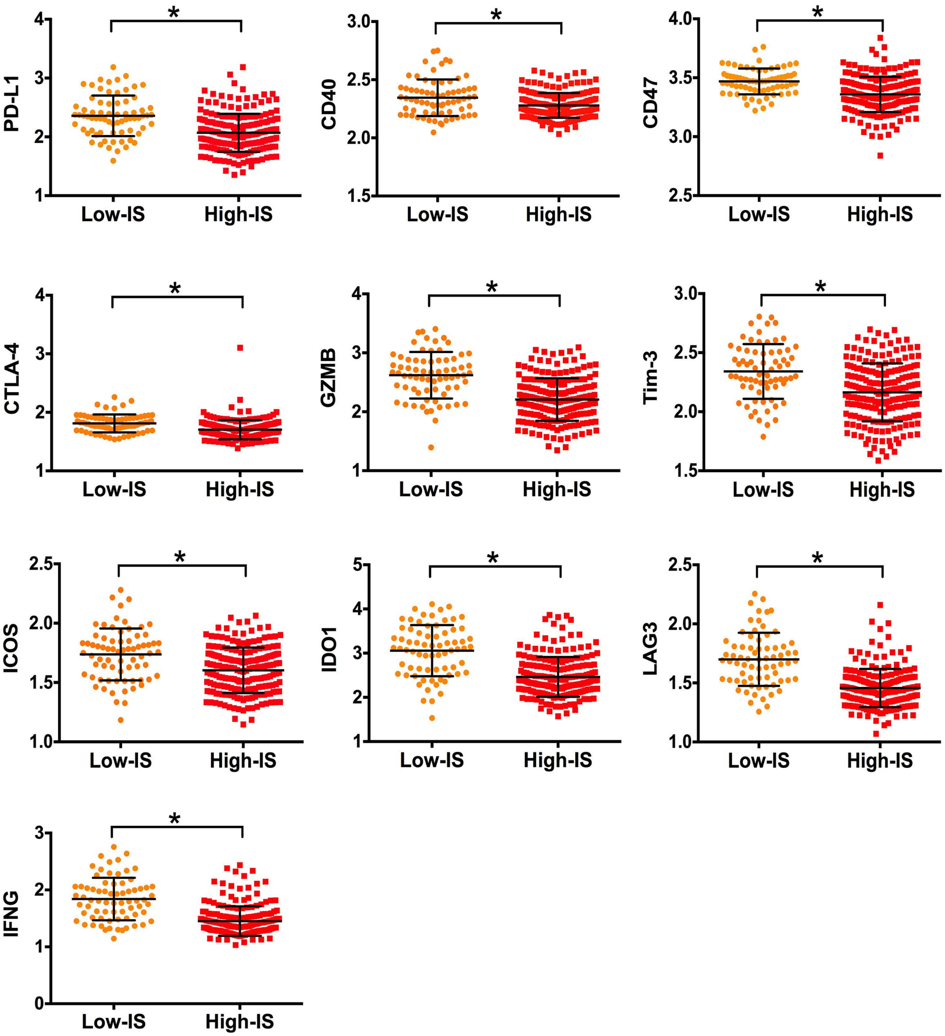
PD-L1
CD40
CD47
CTLA-4
GZMB
Tim-3
ICOS
IDO1
LAG3
IFNG
Low-IS
High-IS
*

Table S1 Univariable and multivariable association of IS classifier, clinicopathological characteristics with overall survival in the training cohort.

	Univariate analysis		Multivariate analysis	
	HR(95% CI)	P value	HR(95% CI)	P value
Gender	0.992(0.822-1.198)	0.937		
Age	1.014(0.997-1.031)	0.113		
T stage(T2+T3 vs. T1+T2)	2.347(1.655-3.327)	<0.01		
N stage(N1+N2+N3 vs. N0)	2.706(1.321-5.543)	0.006		
TNM stage(III vs. I+II)	2.979(2.023-4.387)	<0.01		
Lauren type	1.432(1.008-1.885)	0.01		
Status of microsatellite (MSI vs.MSS)	1.283(1.023-1.608)	0.031		
Liver metastasis	4.195(2.742-6.418)	<0.01	2.378(1.455-3.886)	0.001
Peritoneal metastasis	4.974(3.375-7.331)	<0.01	2.251(1.449-3.499)	<0.01
Bone metastasis	4.664(3.163-6.876)	<0.01	2.585(1.644-4.064)	<0.01
Intraabdominal_LN	2.661(0.843-8.399)	0.095		
IS	1.694(1.425-2.014)	<0.01	1.296(1.042-1.614)	0.02

Abbreviation: *MSS* microsatellite stability; *MSI* microsatellite instability; *HR* hazard ratio; *LN* lymph node; *IS* immunoscore.

Table S2 Univariable and multivariable association of IS classifier, clinicopathological characteristics with disease-free survival in the testing cohort.

	Univariate analysis		Multivariate analysis	
	HR(95% CI)	P value	HR(95% CI)	P value
Gender	0.792(0.545-1.150)	0.221		
Age	0.998(0.982-1.014)	0.82		
TNM (III vs. I+II)	2.574(1.820-3.641)	<0.01	2.599(1.838-3.676)	<0.01
Lauren type	0.975(0.703-1.352)	0.879		
IS	1.926(1.181-3.142)	0.009	1.899(1.200-3.005)	0.006

Abbreviation: *HR* hazard ratio; *IS* immunoscore.

Table S3 Univariable and multivariable association of IS classifier, clinicopathological characteristics with overall survival in the testing cohort.

	Univariate analysis		Multivariate analysis	
	HR(95% CI)	P value	HR(95% CI)	P value
Gender	0.800(0.543-1.177)	0.258		
Age	1.000(0.983-1.017)	0.991		
TNM (III vs. I+II)	2.842(1.983-4.072)	<0.01	2.863(1.997-4.102)	<0.01
Lauren type	1.084(0.773-1.520)	0.64		
IS	2.026(1.224-3.353)	0.006	1.997(1.234-3.167)	0.005

Abbreviation: *HR* hazard ratio; *IS* immunoscore.
